# Parent and child gender effects in the relationship between attachment and both internalizing and externalizing problems of children between 2 and 5 years old: A dyadic perspective

**DOI:** 10.1002/imhj.70002

**Published:** 2025-02-09

**Authors:** Magda Matetovici, Anouk Spruit, Cristina Colonnesi, Mauricio Garnier‐Villarreal, Marc Noom

**Affiliations:** ^1^ Research Institute of Child Development and Education University of Amsterdam Amsterdam The Netherlands; ^2^ Language Development Department MPI for Psycholinguistics, Max Planck Society Nijmegen The Netherlands; ^3^ Basic Trust Dordrecht The Netherlands; ^4^ Faculty of Social Sciences, Sociology Vrije Universiteit Amsterdam Amsterdam Noord‐Holland The Netherlands

**Keywords:** attachment, externalizing problems, gender, internalizing problems, parent–child dyads, التعلق، مشاكل السلوك الداخلي، مشاكل السلوك الخارجي، ، الجنس، ثنائيات الوالدين والطفل, 依恋, 内化问题, 外化问题, 性别, 亲子二元关系, Attachement, problèmes d'internalisation, problèmes d'externalisation, genre, dyades parent‐enfant, Bindung, internalisierende Probleme, externalisierende Probleme, Geschlechtsidentität, Eltern‐Kind‐Dyaden, 愛着、内在化問題、外在化問題、性差、親子ペア, afectividad, problemas de internalización, problemas de externalización, género, díadas progenitor‐niño

## Abstract

Acknowledging that the parent–child attachment is a dyadic relationship, we investigated differences between pairs of parents and preschool children based on gender configurations in the association between attachment and problem behavior. We looked at mother–daughter, mother–son, father–daughter, and father–son dyads, but also compared mothers and fathers, daughters and sons, and same versus different gender pairs. We employed multigroup structural equation modeling to explore moderation effects of gender in a sample of 446 independent pairs of parents and preschool children (2–5 years old) from the Netherlands. A stronger association between both secure and avoidant attachment and internalizing problems was found for father–son dyads compared to father–daughter dyads. A stronger association between both secure and avoidant attachment and externalizing problems was found for mother–son dyads compared to mother–daughter and father–daughter dyads. Sons showed a stronger negative association between secure attachment and externalizing problems, a stronger positive association between avoidant attachment and externalizing problems, and a stronger negative association between secure attachment and internalizing problems compared to daughters. These results provide evidence for gender moderation and demonstrate that a dyadic approach can reveal patterns of associations that would not be recognized if parent and child gender effects were assessed separately.

## ملخص

التعلق بين الوالدين والطفل هو علاقة ثنائية، ولذلك قمنا بدراسة الاختلافات بين أزواج من الآباء والأمهات والأطفال في مرحلة ما قبل المدرسة على أساس خصائص الجنس في العلاقة بين التعلق والسلوك الإشكالي. نظرنا في ثنائيات الأم والبنت، والأم والابن، والأب والبنت، والأب والابن، وقارنا أيضًا بين الأمهات والآباء، والبنات والأبناء، والثنائيات من نفس الجنس مقابل ثنائيات من جنسين مختلفين. استخدمنا نمذجة المعادلات الهيكلية متعددة المجموعات لاستكشاف التأثيرات المتغيرة للجنس في عينة مكونة من 446 زوجًا مستقلاً من الآباء والأمهات والأطفال في مرحلة ما قبل المدرسة (من سنتين إلى 5 سنوات) من هولندا. أظهرت النتائج ارتباطاً أقوى بين كل من التعلق الآمن والتعلق التجنبي والمشاكل الداخلية لثنائيات الأب والابن مقارنة بالثنائيات بين الأب والبنت. كما أظهرت النتائج ارتباطاً أقوى بين كل من التعلق الآمن والتعلق التجنبي ومشاكل السلوك الخارجي في ثنائيات الأم والابن مقارنة بثنائيات الأم والابنة والأب والابنة. وأظهر الأبناء ارتباطاً سلبياً أقوى بين التعلق الآمن ومشاكل السلوك الخارجي، وارتباطاً إيجابياً أقوى بين التعلق التجنبي ومشاكل السلوك الخارجي، وارتباطاً سلبياً أقوى بين التعلق الآمن ومشاكل السلوك الداخلي مقارنة بالبنات. تقدم هذه النتائج دليلاً على ثأثير الجنس وتوضح أن النهج الثنائي يمكن أن يكشف عن أنماط من الارتباطات التي لن يتم التعرف عليها إذا تم تقييم تأثيرات الوالدين والطفل بشكل منفصل.

## 摘 要

考虑到亲子依恋是一种二元关系, 我们研究了依恋与问题行为之间的关联在不同性别配置的父母与学龄前儿童中的差异。我们探讨了母女、母子、父女和父子配对, 同时比较了母亲与父亲、女儿与儿子以及同性与异性配对的情况。研究采用多组结构方程模型, 分析了来自荷兰的446对独立的父母与学龄前儿童 (2至5岁) 样本中性别的调节作用。结果显示, 在父子配对中, 安全型依恋与回避型依恋与内化问题的关联强于父女配对;在母子配对中, 安全型依恋与回避型依恋与外化问题的关联强于母女和父女配对。此外, 与女儿相比, 儿子表现出更强的以下关联:安全型依恋与外化问题之间的负向关联、回避型依恋与外化问题之间的正向关联, 以及安全型依恋与内化问题之间的负向关联。这些结果为性别的调节作用提供了证据, 表明通过二元关系的方法, 可以揭示出单独评估父母和子女性别效应时无法识别的关联模式。

Tout en reconnaissant que l'attachement parent‐enfant est une relation dyadique, nous avons étudié les différences entre les paires de parents et les enfants d’âge préscolaire en fonction des configurations de genre dans le lien entre l'attachement et les problèmes de comportement. Nous avons examiné les dyades mère‐fille, mère‐fils, père‐fille et père‐fils, mais aussi comparé les mères et les pères, les filles et les fils et les mêmes paires de sexes par rapport à des couples de sexe différent. Nous avons utilisé une modélisation d’équations structurelles multi‐groupes pour explorer les effets de modération du sexe sur un échantillon de 446 couples indépendants de parents et d'enfants d’âge préscolaire (âgés de 2 à 5 ans) aux Pays‐Bas. Un lien plus fort entre l'attachement à la fois sécure et évitant et les problèmes d'internalisation a été trouvé pour les dyades père‐fils par rapport aux dyades père‐fille. Un lien plus fort entre l'attachement à la fois sécure et évitant et les problèmes d'externalisation a été trouvé pour les dyades mère‐fils par rapport aux dyades mère‐fille et père‐fille. Les fils ont fait preuve d'un lien négatif plus fort entre l'attachement sécurisé et les problèmes d'externalisation, d'un lien positif plus fort entre l'attachement évitant et les problèmes d'externalisation, et d'un lien négatif plus fort entre l'attachement sécurisé et les problèmes d'internalisation par rapport aux filles. Ces résultats fournissent des preuves de la modération fondée sur le sexe et démontrent qu'une approche dyadique peut révéler des modèles de liens qui ne seraient pas reconnus si les effets de genre parents et enfants étaient évalués séparément.

Da es sich bei der Eltern‐Kind‐Bindung um eine dyadische Beziehung handelt, untersuchten wir Unterschiede zwischen Elternpaaren und Vorschulkindern basierend auf Konfigurationen ihrer Geschlechtsidentität in Bezug auf den Zusammenhang zwischen Bindung und Problemverhalten. Wir untersuchten Mutter‐Tochter‐, Mutter‐Sohn‐, Vater‐Tochter‐ und Vater‐Sohn‐Dyaden, verglichen aber auch Mütter und Väter, Töchter und Söhne sowie Paare gleicher oder unterschiedlicher Geschlechtsidentität. Wir setzten Mehrgruppen‐Strukturgleichungsmodellierung ein, um Moderationseffekte der Geschlechtsidentität in einer Stichprobe von 446 unabhängigen Paaren von Eltern und Vorschulkindern (2 bis 5 Jahre alt) aus den Niederlanden zu untersuchen. Ein stärkerer Zusammenhang zwischen sicherer und vermeidender Bindung und internalisierenden Problemen wurde bei Vater‐Sohn‐Paaren im Vergleich zu Vater‐Tochter‐Paaren festgestellt. Ein stärkerer Zusammenhang zwischen sicherer und vermeidender Bindung und externalisierenden Problemen wurde bei Mutter‐Sohn‐Dyaden im Vergleich zu Mutter‐Tochter‐ und Vater‐Tochter‐Dyaden festgestellt. Söhne zeigten einen stärkeren negativen Zusammenhang zwischen sicherer Bindung und externalisierenden Problemen, einen stärkeren positiven Zusammenhang zwischen vermeidender Bindung und externalisierenden Problemen und einen stärkeren negativen Zusammenhang zwischen sicherer Bindung und internalisierenden Problemen als Töchter. Diese Ergebnisse liefern Belege für eine bezüglich der Geschlechtsidentität spezifische Moderation und zeigen, dass ein dyadischer Ansatz Assoziationsmuster aufdecken kann, die nicht erkannt würden, wenn geschlechtsspezifische Effekte nach Eltern und Kindern getrennt untersucht würden.

親子の愛着は二者関係とされており、愛着と問題行動との関連における、性別構成に基づく親と未就学児のペア間の差異を検証した。母‐娘、母‐息子、父‐娘、父‐息子のペアに注目したが、母親と父親、娘と息子、同性のベアと異性のペアも比較した。オランダの446組の独立した親と未就学児 (2～5歳) のサンプルを用いて、多群構造方程式モデリングを用い、性別の調整効果を調べた。安定型愛着と回避型愛着の両方と内在化問題との間には、父娘ペアに比べて父子ペアでより強い関連が認められた。安全愛着と回避性愛着の両方と外在化問題との間には、母‐娘、父‐娘ペアと比較して、母‐息子ペアでより強い関連が認められた。息子は娘に比べて、安定型愛着と外在化問題の間に強い負の関連、回避型愛着と外在化問題の間に強い正の関連、安定型愛着と内在化問題の間に強い負の関連を示した。これらの結果は、性差による調整の証拠を示し、親子の性差の影響を別々に評価した場合には認められないような関連性のパターンを、二者アプローチによって明らかにできることを示している。

## Resumen

Reconociendo que la unión afectiva progenitor‐niño es una relación diádica, investigamos las diferencias entre pares de progenitores y niños prescolares con base en configuraciones de género en la asociación entre afectividad y comportamiento problemático. Consideramos las díadas mamá‐hija, mamá‐hijo, papá‐hija y papá‐hijo, pero también comparamos mamás y papás, hijas e hijos, así como los pares del mismo género versus de diferente género. Empleamos un modelo de ecuación estructural de multigrupo para explorar efectos de moderación de género en un grupo muestra de 446 pares independientes de progenitores y niños prescolares (de 2 a 5 años) de Holanda. Se notó una más fuerte asociación entre ambas, la afectividad segura y afectividad esquiva, y los problemas de internalización en el caso de las díadas papá‐hijo tal como se les comparó con las díadas papá‐hija. Se notó una más fuerte asociación entre ambas, la afectividad segura y la afectividad esquiva y los problemas de externalización en el caso de las díadas mamá‐hijo tal como se les comparó con las díadas mamá‐hija y papá‐hija. Los hijos mostraron una más fuerte asociación negativa entre la afectividad segura y los problemas de externalización, una más fuerte asociación positiva entre la afectividad esquiva y los problemas de externalización, y una más fuerte asociación negativa entre la afectividad segura y los problemas de internalización, tal como se les comparó con las hijas. Estos resultados proveen evidencia para la moderación de género y demuestran que un acercamiento diádico puede revelar patrones de asociaciones que so serían reconocidos si los efectos del género del progenitor y del niño fueran evaluados separadamente.

## 1 Introduction

Research on the association between attachment and children's mental health has historically been focused on mothers (Bretherton, 2010; Schuengel et al., 2021), most likely because they are often the primary caregivers, spending more time with children than other family members (Craig & Mullan, 2011; Pakaluk & Price, 2020). Moreover, studies have mainly focused on either the parent or the child in the parent–child relationship, overlooking the fundamental nature of attachment as a dyadic relationship formed between one parent and one child (Ainsworth et al., 1978; Bowlby, 1969; Cassidy & Shaver, 2002; Sroufe, 2005; Zeanah & Boris, 2000). Yet, attachment depends on both the parent and the child (George & Solomon, 2008; Powell et al., 2013; Rothbaum et al., 2002).

In recent decades, the increased involvement of fathers in childcare (Cabrera et al., 2000) has reoriented researchers’ attention to the role of paternal attachment (e.g., Cabrera, 2020; Grossmann & Grossmann, 2020) and has raised the question of whether boys and girls respond differentially to the attachment with their caregivers (e.g., Kamza, 2019). While the importance of gender in attachment relationships is sometimes acknowledged (Deneault et al., 2021; Fearon et al., 2010; Hoeve et al., 2012; Madigan et al. 2013), the parent–child gender interaction is rarely considered and deserves more scrutiny. Thus, this paper aimed to reduce the knowledge gap regarding how attachment to mothers and fathers specifically relates to their daughters’ or sons' mental health during early childhood by offering a detailed analysis at the dyadic level.

**Key findings**

- In father‐son dyads, there was a stronger negative association between secure attachment and internalizing problems and a stronger positive association between avoidant attachment and internalizing problems compared to father–daughter dyads.
- In mother–son dyads, there was a stronger negative association between secure attachment and externalizing problems and a stronger positive association between avoidant attachment and externalizing problems compared to both mother–daughter and father–daughter dyads.
- Dyads with sons exhibited a stronger negative association between secure attachment and externalizing problems, a stronger positive association between avoidant attachment and externalizing problems, and a stronger negative association between secure attachment and internalizing problems compared to dyads with daughters.

**Statement of Relevance**

The results of this study indicate that the strength of the bidirectional influence between some attachment patterns (secure and avoidant) and problem behavior (internalizing and externalizing problems) differs between parent–child dyads based on the gender of the parent and the gender of the child. They motivate the adoption of a dyadic approach in addition to assessing the role of parent and child gender separately in future studies looking at the association between attachment and problem behavior.

### 1.1 Attachment and psychopathology

In early childhood, parents are typically the strongest attachment figures for children, and they have the biggest impact on their psychological well‐being (DePasquale & Gunnar, 2020; Bosmans & Kerns, 2015; Bretherton, 1992; Lamb & Lewis, 2013; Malekpour, 2007; Ranson & Urichuk, 2008). Because of this powerful influence, the potential impact of a low attachment quality on psychopathology has been an area of active research interest (Bretherton, 1985; Fearon & Roisman, 2017; Kochanska, 2001; Schuengel et al., 2021; Sroufe, 2005; Slater, 2007).

A secure attachment is a strong connection formed when a relationship proves to be a constant source of emotional and physical security, and it is reflected in a healthy balance between seeking proximity to the caregiver and distancing from them to explore the environment independently (Bowlby, 1969, 1973, 1980). There are several explanations as to why attachment would impact the mental health of children. An insecure attachment means that the child worries about the caregivers’ availability or feels that they have failed to maintain this vital connection, predisposing them to symptoms of anxiety and depression (Bowlby, 1973; 1980). Conversely, a secure attachment relationship is a buffer against stressors through its impact on children's emotional regulation, potentially protecting them from developing mental health problems (Cassidy et al., 2013; Cooke et al., 2019; Schore, 2001). Moreover, a secure attachment makes children more receptive to norms and values transmitted by their parents – leading to fewer conduct problems as argued by Kochanska et al. (2004).

The proposition that attachment is associated with children's psychosocial development has now been tested in numerous studies (Ranson & Urichuk, 2008). These studies usually conceptualize children's mental health through Achenbach's (1966) division into internalizing and externalizing problems. For example, shyness, worries, nightmares, or excessive tiredness are internalizing problems, while cruelty, destructive behavior, lying, or fighting are externalizing problems (Achenbach, 1966). In terms of attachment, studies compare secure to insecure attachment, the latter being broken down into two discrete categories widely used in research: insecure‐avoidant and insecure‐resistant/ambivalent (Ainsworth et al., 2015; Benoit, 2004; Powell et al., 2013; Solomon & George, 1999). In addition, a distinction is made between organized attachment (comprising of secure and insecure attachment) and disorganized attachment (Hesse & Main, 2000; Main & Solomon, 1986) based on the existence or absence of a consistent, predictable attachment strategy. In short, an avoidant attachment is characterized by a tendency to keep distance from the caregiver and to show indifference toward them (Ainsworth, 1985; Benoit, 2004). Ambivalent attachment manifests through an alternation between the child being overly dependent on their parent and resisting contact with them (Ainsworth et al., 1985; Benoit, 2004). Disorganized attachment is characterized by a lack of regularity in the parent–child relationship due to the fact that children both seek comfort from and are afraid of their caregivers, and it is often associated with harmful or inappropriate parenting behavior (Benoit, 2004; Main & Solomon, 1986).

### 1.2 Attachment and internalizing problems

Brumariu and Kerns (2010) offered the first review of studies looking at parent–child attachment and internalizing problems during childhood. Almost all of the reviewed studies concerned maternal attachment to children between 2 and 10 years old, apart from one about paternal attachment. The authors identified studies that found no effect as well as studies that found a negative effect of attachment security on internalizing problems. They noticed that the effects were more consistent when anxiety and depression were measured rather than global internalizing problems. Indeed, this remark has been supported by subsequent studies. Two meta‐analyses that looked specifically at anxiety symptoms (Colonnesi et al., 2011) and depressive symptoms (Spruit et al., 2020) from early childhood to adolescence found medium‐size effects concerning attachment: *d* = .62 and *d* = .65, respectively. Weaker and more varying effect sizes were found by meta‐analyses looking at global internalizing problems: *d* = .15 (Groh et al., 2012), *d* = .15 (Groh et al., 2017), *d* = .19 (Madigan et al., 2013), and *d* = .58 (Madigan et al., 2016). The only meta‐analysis on paternal attachment and internalizing problems also found a small effect size similar to studies that largely assess maternal attachment: *d* = .17 (Deneault et al., 2021). These varying results could be attributed to different age ranges studied – a possibility highlighted by the systematic review of Badovinac et al. (2021) where a secure early attachment was consistently associated with fewer preschool internalizing problems, but not with fewer pre‐adolescent internalizing problems. This is, however, not consistent with a recent study in infants that found no association between attachment to parents and internalizing problems (Umemura et al., 2022). Interestingly, in a recent individual participant data meta‐analysis no difference in internalizing problems was found between children insecurely attached to one parent and children insecurely attached to both parents, but children securely attached to both parents had fewer internalizing problems compared to the first two groups (Dagan et al., 2021). Moreover, when the report of internalizing problems was averaged between mothers and fathers, no difference was found between insecurely attached and securely attached children (Dagan et al., 2021). This suggests that when it comes to internalizing problems, secure attachment to parents might be additive while insecure attachment might not be – another explanation for the mixed results found in other studies.

### 1.3 Attachment and externalizing problems

Besides internalizing problems, insecurely attached children also seem to have more externalizing problems. This effect is slightly more robust than in the case of internalizing problems: *d* = .37 (Deneault et al., 2021), *d* = .31 (Groh et al., 2017), *d* = .31 (Fearon et al., 2010), *d* = .37 (Hoeve et al., 2012), and *d* = .49 (Madigan et al., 2016). Again, the evidence comes from studies that generally assess the attachment with the mother, with the exception of Deneault et al.’s (2021) meta‐analysis on paternal attachment. As in the case of internalizing problems, more consistent results are found when looking at preschool children (Badovinac et al., 2021). This is in line with two meta‐analyses on studies with participants between 3 and 18 (Madigan et al., 2016) and between 6 and 38 years old (Hoeve et al., 2012) which found that the strength of the association decreases with age, thus stronger effects are seen in younger participants. Notably, Dagan et al. (2021) also found a relation between parent–child attachment and externalizing problems when only mothers reported problems but found no difference between groups of children who had secure versus insecure attachment when the report of externalizing problems was averaged between the two parents.

### 1.4 Problem behavior and subtypes of insecure attachment

Overall, review and meta‐analytic evidence indicate that, when researchers compare a secure versus an insecure attachment, there is a small to medium association with internalizing and externalizing problems (Badovinac et al., 2021; Brumariu & Kerns, 2010; Colonnesi et al., 2011; Deneault et al., 2021; Groh et al., 2017; Fearon et al., 2010; Hoeve et al., 2012; Madigan et al., 2013; Madigan et al., 2016; Spruit et al., 2020), although a minority of studies also fail to find this association (Dagan et al., 2021; Umemura et al., 2022). When studies look at the sub‐types of insecure attachment, however, the results are less conclusive.

In the review done by Brumariu and Kerns (2010), for children under the age of 10 insecure‐ambivalent attachment was associated with greater anxiety compared to the other insecure types, while insecure‐disorganized attachment was most consistently linked to global internalizing problems. Interestingly, Madigan et al. (2013) found that only insecure‐avoidant attachment was associated with more internalizing behavior while insecure‐ambivalent and insecure‐disorganized attachment were not in children between 1.5 and 10 years of age, similar to what Groh et al. (2012) found in studies with samples of children under 18. In contrast, Madigan et al. (2016) found that all subtypes of insecure attachment related positively to internalizing problems, while only insecure‐disorganized attachment related positively to externalizing problems when looking at studies with children between 3 and 18 years old. Insecure‐disorganized attachment has been previously shown to be associated with more externalizing problems in infants (van IJzendoorn et al., 1999) and more internalizing problems in children (Groh et al., 2012). Fearon et al. (2010) found that, while insecure attachment is generally associated with more externalizing problems, the effect size is bigger for insecure‐disorganized compared to insecure‐ambivalent and insecure‐avoidant attachment in children below 12 years of age. These results show that the relation between insecure‐disorganized and externalizing problems is the most robust, while there are still inconsistencies in terms of the other two subtypes’ (i.e., avoidant and ambivalent) associations with internalizing and externalizing problems.

### 1.5 Gender roles

While the relation between attachment and mental health is very prominent in attachment research, no study, to our knowledge, had the main goal of examining whether the association between attachment and behavioral problems is different for dyads resulting from combining the gender of the parent and the child, such as mother–daughter dyads, mother–son dyads, father–daughter dyads, and father–son dyads, as well as in same versus different gender dyads. Gender is worth investigating for several reasons. First, there is a biased focus in the literature on mother‐child attachment, neglecting the importance of father–child attachment, especially as the father's role in child‐rearing becomes more prominent after infancy (Bianchi, 2000; Lamb, 2004; Pleck et al., 2004). Second, gender is relevant when considering the mutual interactions between parents and children (Hinde & Hinde, 1988). As children become aware of their gender in early childhood (Leaper & Friedman, 2007; Martin et al., 2002; Martin & Ruble, 2010) and start preferring same gender play partners (Hoffmann & Powlishta, 2001), gender might be a salient grouping factor inside families (e.g., Nikiforidis et al., 2018). Gender has been considered important in family dynamics (Dumont & Paquette, 2013; Maccoby, 1990; Moon & Hoffman, 2008; Siegal, 1987).

Several authors considered the possibility that mothers and fathers play different roles in children's upbringing, as a basis to argue that gender plays a role in the impact parents have on children. Mothers have been pictured as those who provide emotional support, comfort, and reassurance (Starrels, 1994; Verschueren, 2020), while fathers have been pictured as the ones that teach discipline, provide practical support (Starrels, 1994), and encourage children to explore and gain autonomy (Bögels & Phares, 2008; Verschueren, 2020).

Still, as men and women adopt less traditional roles and share more of the child‐rearing responsibilities (Marshall, 2006), it is possible that more recent studies see fewer differences in how mothers and fathers interact with their children. Grossmann et al. (2008) also point out this shift in the roles of fathers and mothers – in the past, they used to be more distinct than they are today, so both parents may have a similar impact on developmental outcomes, with mothers and fathers equally providing emotional support as well as encouragement to discover the environment.

Another possibility is that gender can enhance the effect of a good parent–child relationship through the fact that gender is a shared trait. The shared trait of gender between a parent and a child could facilitate mutual understanding, enhance interaction, and promote mutual influence. This assertion is in line with gender‐based homophily, a phenomenon largely studied in peer relationships and other social interactions (McPherson et al., 2001), but, so far, not in parent‐child interactions. Yet, there are studies that investigated a related, perhaps overlapping effect: the similarity effect. This effect entails that having the same gender will translate into a stronger relationship and a stronger effect of this relationship on the outcomes of children (Russell & Saebel, 1997; Xu et al., 2018). It can be explained by the fact that children are more likely to observe and imitate the behavior of those who seem similar to them (social learning theory, Bandura & Walters, 1977) or by the fact that when people perceive themselves as similar, they communicate better and form stronger relationships (similarity‐attraction theory, Newcomb, 1956). This is in line with Nikiforidis’ (2018) finding that mothers spend relatively more time with daughters and fathers with sons and that this choice is mediated by parents’ identification with their children, and partially in line with Raley and Bianchi's (2006) finding that fathers spend more time with sons, but mothers spend equal amounts of time with their children.

To our knowledge, only the review study by Russell and Saebel (1997) explored the claim that parent–child gender interactions could have widespread effects. The review contains studies on children ranging from infancy to adolescence and includes research pertaining to the role of gender specifically, but also studies that included gender in supplementary analyses (it is, however, limited to the studies published in four journals between 1990 and 1993). It considers a variety of indicators of the parent–child relationship such as closeness, emotional support, or parenting style, as well child outcomes associated with the parent–child relationship such as self‐esteem or attitude toward parents. Various patterns of differences in the four dyads were considered, for example, that they are all distinct, that one of them is distinct, or that only the parent's or child's gender determines differences (see table 1 in Russell & Saebel, 1997). Of relevance to this paper, the authors could identify only one study which explored the role of gender with regard to the outcomes associated with parent–child attachment – a study that showed attachment to mothers and not to fathers was associated with identity achievement in late adolescents (Benson et al., 1992). Drawing upon the mixed and weak effects discovered, Russell and Saebel (1997) reached the conclusion gender may contribute to differences, but these effects are not seen across all possible child outcomes. However, they noticed that when differences were found, it was in studies that looked at emotional aspects of the relationship such as closeness, cohesion, and affection. This motivates investigating gender effects interactions in the case of attachment, a construct that involves these emotional aspects (Bell et al., 1998).

### 1.6 Gender, the parent–child relationship and mental health

Specifically in studies about the impact of the parent–child relationship on the mental health of children, gender has often been considered a moderator (Bradley & Corwyn, 2013; Brumariu & Kerns, 2010; Groh et al., 2017; Hoeve et al., 2012; Kochanska et al., 2004; Madigan et al., 2013; Spruit et al., 2020), but mixed findings have been accumulating. In this stream of research, studies can be grouped into those who look at parents’ gender and those who look at children's gender.

In a clinical sample of children between 9 and 16 years old, Mathijssen et al. (1998) found that the gender of the parent moderated the impact of four dimensions of dyadic family relationship quality (restrictiveness, justice, recognition, and trust) on problem behavior. When the mother–child relationship was more restrictive, with lower fairness, trust, and recognition, children exhibited more externalizing problems. When the father–mother relationship was lower on fairness, trust, and recognition, children exhibited more internalizing problems. The father–child relationship quality as reflected by these dimensions was not associated with either internalizing or externalizing problems in the sample of children studied. Attachment to parents as reported by 6‐ to 12‐year‐old children was found to have different associations with children's mental health reported by parents and teachers, depending on parents’ gender (El‐Sheikh & Buckhalt, 2003). Father‐child attachment, not mother–child attachment was associated with childhood internalizing problems, while attachment to both parents related to externalizing problems (El‐Sheikh & Buckhalt, 2003). Recently, however, a meta‐analysis across nine attachment studies on toddlers with a mean age of 2.5 years old found no differences between the attachment to fathers or mothers in terms of insecure/secure attachment and its impact on internalizing and externalizing problems (Dagan et al., 2021).

Similar mixed findings are also reported in studies that focus on children's gender. Madigan et al. (2013) revealed in their meta‐analysis that in boys there was a significant positive relation between insecure attachment and internalizing behavior but not in girls, in samples of infants and toddlers. However, Spruit et al. (2020) found that the relation between attachment and depression is stronger for girls than for boys, where the age range studied was 0–23. In contrast, a meta‐analysis by Groh et al. (2017) on a similar age range found no gender differences in the impact of insecure attachment on internalizing problems, but on externalizing problems the association between attachment insecurity and externalizing problems was stronger for boys than for girls. A stronger association of externalizing problems with attachment insecurity for boys was also found by Fearon et al. (2010) in a sample of children under 12 years old. Of interest, Browne et al. (2010) found that gender moderates the relations between parenting and psychopathology. They showed that the relation between parental inconsistency and anxiety was stronger for boys than girls and that parental hostility did not lead to more aggressive behaviors for one child gender over the other. While the relations between attachment and problem behaviors seems to be stronger for boys, Brock and Kochanska (2016) found that marital conflict predicts internalizing problems by decreasing the attachment security between parents and girls but not by decreasing the attachment security between parents and boys.

Only one study so far has investigated the differences between the four family dyads in terms of the association between the quality of their relations and young children's mental health (Xu et al., 2018). This study looked at closeness and conflict with parents. The results showed only limited evidence for the similarity effect in terms of the moderating role of gender on the impact of closeness and conflict on problem behavior. The mother–daughter closeness predicted less externalizing but not internalizing problems, while mother–daughter conflict and both father–daughter closeness and conflict did not predict either. The father–son conflict predicted more internalizing and externalizing problems, but father–son closeness did not predict either, and neither did mother–son closeness and conflict. These results are only partially consistent with Hoeve et al.’s (2012) meta‐analysis, which revealed stronger effects of attachment on delinquency in same‐gender dyads compared to opposite‐gender dyads. However, their sample included both children and adults, with a maximum age of 38 years old. Reconciling these results is difficult because the studies have both different age ranges and have studied different aspects of the parent–child relationship. Yet, if a similarity effect exists, it is puzzling why it would exist for only one same‐gender pair or only for specific child outcomes, as the study by Xu et al. (2018) seems to indicate.

### 1.7 Current study

Gender has been receiving attention in the literature looking at parent–child relationships and mental health, but looking at both genders in the parent–child dyad has been done rarely. There is evidence supporting an association between attachment to parents and both internalizing and externalizing problems in early childhood, but no study has directly explored potential differences between different parent–child gender configurations in terms of this relation. Until now, studies have been assessing the moderating role of gender for parents and children separately in attachment studies, with mixed results. Therefore, in the current study, we propose a detailed analysis of potential moderating effects of gender by looking at the dyads and by considering different attachment quality patterns (secure, ambivalent, avoidant, and disorganized).

The goal of this study was to investigate the moderating role of gender on the contemporaneous association between attachment and internalizing and externalizing problems in the age group 2–5 years old, an age range that has received less attention in the attachment literature compared to infancy and adolescence. Because of the scarce literature, mixed findings, and lack of a strong theoretical foundation to explain parent–child gender effects, the analyses presented in this paper remain exploratory. Thus, the research question we explored was “*Are there gender differences in terms of the association between attachment and both internalizing and externalizing problems*?”. Specifically, we explored whether the four parent–child gender configurations, parent gender, child gender and parent–child gender similarity moderate the relation between attachment and problem behavior.

## 2 METHODS

### 2.1 Data source and demographics

The dataset used in this study contained information from 509 parents and was collected between 2017 and 2019 in the Netherlands by Spruit et al. (2021). A total of 378 participants were recruited from the general population through social media, child‐care facilities, and schools. Sixty‐eight participants were recruited from the clinical population through collaboration with several organizations for youth care and child psychiatry. All participants signed an informed consent form for participating in the study. In the total sample, 63 pairs of parents reported about the same child. To maintain the independence of observations, Spruit et al. (2021) randomly excluded observations from one caregiver in each parent pair. All caregivers in the sample were the children's parents, with 93.5% being biological parents and 6.5% being adoptive, foster‐ or step‐parents. Most of the parents (88%) were married or living together with their partner, 6% were single parents, 5% were divorced, and 1% were living in another arrangement.

About half of the parents reported about girls (*N* = 221) and half reported about boys (*N* = 225). Children had a mean age of 3.74 (*SD* = 1.11) years. Parents had a mean age of 35.82 (*SD* = 6.00) years. There were 234 mothers and 212 fathers. There were 118 mother–daughter, 116 father–daughter, 103 mother–son, and 109 father–son dyads. The sample was diverse in terms of parents’ education (7% high‐school, 24% secondary vocational education, 38% higher professional education, and 31% university) and the number of children in the family (one child 30%, two children 50%, three children 13%, and four or more children 7%).

### 2.2 Instruments

#### 2.2.1 Demographic information

Parents reported their age, the age of their children and the number of children in their family through an open question. They reported the gender of their children and their own gender through multiple choice questions with options female, male, and other. They reported their education through ten categories based on the Dutch educational system ranging from no education to university education. For this analysis the categories were collapsed to four levels: high school, secondary vocational education, higher professional education, and university.

#### 2.2.2 ARI‐CP 2–5

The Attachment Relationship Inventory‐Caregiver Perception 2–5 years (ARI‐CP 2–5, Spruit et al., 2021) was used to measure parents’ perception of their children's attachment. The ARI‐CP 2–5 is a questionnaire containing 48 statements about the parent–child attachment relationships which can be evaluated on a 5‐point Likert scale in terms of how much they apply to the respondent (1 = does not apply, 5 = fully applies). An example of such a statement is “*My child and I like to cuddle each other*”. There are four subscales, as revealed by confirmatory factor analysis, and these belong to the classic attachment categories present in the literature: Secure (13 statements), Avoidant (11 statements), Ambivalent (11 statements), and Disorganized (13 statements). The scores for each statement can be summed to obtain a total score for each subscale. For this paper's dataset, the questionnaire was administered in Dutch. Internal consistency, concurrent, convergent, and predictive validity, relative to established observational measures such as the Attachment Q‐Sort (Waters & Deane, 1985) and the Emotional Availability Scales (EAS, Biringen, 2014), were shown to be satisfactory by Spruit et al. (2021).  In this dataset, the ordinal Cronbach's alpha was 89 for the secure scale, .77 for the avoidant scale, .85 for the ambivalent scale, and .89 for the disorganized scale. Factor and item reliability of the ARI‐CP 2–5 based on our sample are presented in Appendix B for each group of parent–child dyads. High factor reliability was observed for all dyads with respect to secure, ambivalent and disorganized attachment. For avoidant attachment, high reliability was observed in dyads with mothers, but poorer reliability was observed in dyads with fathers. Across the four groups, item reliability was overall satisfactory.

#### 2.2.3 Strengths and difficulties questionnaire

The strengths and difficulties questionnaire (SDQ) (Goodman, 2001) for children between 2 and 4 years of age was used to measure children's internalizing and externalizing problems, as reported by their parents. This questionnaire consists of a list of 25 psychological attributes that children might possess, for instance: “*kind to younger children*” or “*many fears, easily scared*”. The parents report the presence of these attributes on a 3‐point Likert scale with the options: not true (0), somewhat true (1), and certainly true (2). Total scores on five subscales (emotional symptoms, conduct problems, hyperactivity/inattention, peer problems, and prosocial behavior) can be obtained by adding the corresponding items. For the scope of this paper, we estimated the latent variables: externalizing problems (conduct problems and hyperactivity/inattentiveness scales) and internalizing problems (emotional symptoms and peer problems scales) (Goodman et al., 2010). SDQ's satisfactory reliability and validity has been established in several studies (Bergström & Baviskar, 2021; Croft et al., 2015; Goodman, 2001; Muris et al., 2003; Stone et al., 2010). In this dataset, the ordinal Cronbach's alpha for the internalizing scale was .83 and for the externalizing scale was .87. Factor and item reliability of the SDQ based on our sample are presented in Appendix B for each parent‐child dyad. High factor reliability was observed for all dyads. Across the four groups, item reliability was overall satisfactory.

### 2.3 Analysis plan

This study was a secondary analysis of data collected with the initial goal of validating the Attachment Relationship Inventory—Caregiver Perception 2–5 years (ARI‐CP 2–5) (Spruit et al., 2021), an attachment questionnaire suitable for children between 2 and 5 years old, which emphasizes the dyadic nature of attachment. Ethical approval has been granted for this re‐analysis by the University of Amsterdam Ethical Committee (2020‐CDE‐11822). Spruit et al. (2021) have already presented a negative association between attachment security and internalizing and externalizing problems based on these data, at the level of the entire sample. They have also confirmed the measurement invariance of the ARI‐CP 2–5 between mothers and fathers and between boys and girls.

We employed multigroup structural equation modeling to investigate the relation between the four attachment quality types (secure, insecure‐avoidant, insecure‐ambivalent, and insecure‐disorganized) and internalizing and externalizing problems. We chose this technique because it accounts for measurement error better than approaches that use sum scores of the items, giving us more power to detect effects (Kline, 2023). The analysis was performed in R with the Lavaan, semTools, and Mice packages (Jorgensen et al., 2018; Rosseel, 2012; Van Buuren and Groothuis‐Oudshoorn, 2011).

We started by checking the measurement invariance between the four dyads on the six latent variables: four attachment types and two problem behavior domains. We checked for configural, threshold, and metric invariance. For comparing the strength of a relation between two latent variables in different groups, only weak invariance is necessary (Gregorich, 2006). We defined a multigroup structural equation model to evaluate the differences in correlations between groups for the relation between each attachment quality pattern and both internalizing and externalizing problems independently. Overall model fit was evaluated with the approximate fit indices CFI, Gamma‐hat, and SRMR, as these have shown to be more reliable measures of misfit than other indices (Garnier‐Villarreal & Jorgensen, 2020; Fan & Sivo, 2007). We interpret fit indices as descriptive measures of approximate fit, and to interpret questionable values as indicating not that the model should be rejected outright but that further investigation of local sources of misspecification (e.g., correlation of residuals or modification indices) would be warranted. Fit indices were not proposed to function as test statistics but rather to “provide important adjunct information in evaluating models” (Bentler & Bonett, 1980, p. 604). Thus, similar to measures of effect size in other contexts (e.g., Cohen, 1992, provided guidelines to interpret correlations, standardized mean differences, and proportions of variance explained as small, medium, or large), guidelines were sought for interpreting the magnitude of fit indices (Garnier‐Villarreal & Jorgensen, 2020). We evaluate the fit indices as they should approximate the ideal values, and the interpretation and magnitude of the parameters of the measurement model (like factor loadings), but we will not use arbitrary cut‐off values for the fit indices.

As an effect size of the moderation of gender, we used correlations between phantom latent factors to obtain a standardized estimate of the correlation between the latent factors which accounts for differences in latent factors variances between groups, such that their relation is expressed on a common scale (Little, 2024, Chapter 4, pp. 132–137). We analyzed differences at the level of the four dyads, but also compared mothers and fathers, daughters and sons and similar versus different genders in the parent–child dyad. For each problem behavior we performed a total of nine comparisons, therefore we adjusted the *p*‐values using false discovery rate adjustment before evaluating significance. The code used for pre‐processing and analysis can be found at: https://github.com/MagdaMatetovici/AttachmentProblemBehavior.

### 2.4 Data inspection

Upon inspection of the data, several items on the ARI‐CP 2–5 and the SDQ had a frequency of zero for some of the response options in one or more of the dyads. For example, on the item “*I immediately understand what my child needs*” none of the mother‐son pairs chose the option “*not at all applicable*”. In order to use the Weighted Least Square Mean and Variance Adjusted (WLSMV) estimator, suitable for categorical data but only if frequencies for each category are non‐zero, we collapsed categories by merging adjacent categories when one or more of them had zero frequencies as discussed by DiStefano et al. (2021). The response options for which, in some groups, the response frequency was zero were the ones at the extremes of the scale. This is because behaviors associated with the extremes of the scale are rare in the population. Checking whether zero frequencies were present was done at the level of the dyads. For the ARI‐CP 2–5 questionnaire, collapsing was necessary for 42 of the 48 items, resulting in 12 items with three response levels and 30 items with four response levels. For the SDQ questionnaire, collapsing was necessary for four items where the response levels were reduced from 2 to 3.

Our dataset had a trivial amount of missing data points (*N* = 5) compared to the sample size (*N* = 446): four in the ARI‐CP 2–5 questionnaire and one in the SDQ questionnaire. Therefore, a simple stochastic regression imputation was applied.

## 3 RESULTS

### 3.1 Demographic differences between the dyads

We checked whether the four dyads differ in terms of parent education, number of children in the family, child age, and parent age. A Chi‐square test of independence was performed between the categorical variable education (with the Dutch education levels high‐school, secondary vocational education, higher professional education, and university) and the categorical variable dyad (with levels mother–son, mother–daughter, father–daughter, and father–son). The relation between these variables was not significant: $\chi^{2}$ (9, 446) = 12.72, *p* = .180, meaning there is not enough evidence to conclude the dyads are different in terms of education. A second Chi‐square test of independence was performed between the categorical variable number of children in the family (with the levels 1, 2, 3, and 4 and more children) and the categorical variable dyad. The relation between these variables was not significant: $\chi^{2}$ (9, 446) = 11.77, *p* = .230, meaning there is not enough evidence to conclude the dyads are different in terms of the number of children in the family. A one‐way between subjects analysis of variance (ANOVA) revealed there is not enough evidence to conclude the dyads are different in terms of child age: *F*(3, 442) = 2.12, *p* = .097. However, a one‐way between subjects ANOVA indicated a difference between dyads in terms of parent age: *F*(3, 441) = 5.045, *p* = .002. Post hoc Tukey HSD comparisons revealed evidence that the mothers in mother–son dyads (*M* = 34.2, *SD* = 5.33) were younger than fathers in father–son (*M* = 37.10, *SD* = 5.98) and father–daughter dyads (*M* = 36.5, *SD* = 5.67), with a difference of −2.88 years, *p* = .002 and of −2.34 years, *p* = .02, respectively. Consequently, we added parent age as a covariate in the multigroup SEM model, in order to evaluate differences in terms of attachment and problem behavior while controlling for it.

### 3.2 Correlations among latent variables

Table 1 displays the correlations between the latent variables of interest at the level of the entire sample. All correlations are statistically significant and have the theoretically expected direction: secure attachment is negatively associated with problem behavior, while insecure attachment is positively associated with problem behavior. Because of the high correlations among the constructs of interest, we evaluate the moderation as the differences between each pairwise (latent) correlation across gender groups. This provides differences in the strength of the relation (moderation) between factors in a standard (easy to understand) metric.

**TABLE 1:** Correlations among latent variables.

	1.	2.	3.	4.	5.
Problem Behavior					
1. Internalizing					
2. Externalizing	.59				
Attachment					
3. Secure	−.58	−.56			
4. Avoidant	.42	.51	−.78		
5. Ambivalent	.70	.71	−.69	.61	
6. Disorganized	.62	.83	‐.65	.70	.92

*Note*: All correlations are statistically significant at *α* < .001.

### 3.3 Measurement invariance

First, measurement invariance between the dyads was checked for the ARI‐CP 2–5 questionnaire and for the internalizing and externalizing problems scales from the SDQ. Fitting the configural models revealed satisfactory fit in terms of the CFI scaled, Gamma‐Hat scaled and SRMR indices (Bentler & Bonett, 1980; Browne & Cudeck, 1992; Hu & Bentler, 1999) for secure, ambivalent, disorganized attachment, and externalizing problems. For the avoidant attachment model and for the internalizing problems model, modifications were made in order to improve the configural fit (Appendix A). Table A3 in the Appendix presents the values for the approximate fit indices.

Next, we tested the invariance of thresholds, loadings and intercepts for each construct (Svetina et al., 2020). Table A2 displays the results for the attachment multigroup CFA models. Table A3 displays the results for the problem behavior multigroup CFA models. Because the SDQ has items with three and two response options, threshold invariance cannot be tested alone as it is already satisfied (Wu & Estabrook, 2016). For the SDQ questionnaire we directly compared the configural to the loadings and thresholds model in order to assess weak measurement invariance.

Full weak measurement invariance was reached for disorganized and externalizing attachment (Tables A1 and A2). Partial weak measurement invariance was reached for secure, avoidant, ambivalent attachment and for internalizing problems following model modifications (Tables A1 and A2). Tables A3 and A4 show the relative fit indices for all models ran to assess measurement invariance. Appendix A describes the modification made for achieving partial measurement invariance.

### 3.4 Main analysis

We ran two multigroup SEM models to assess differences in the relation between problem behavior and attachment in terms of gender: one model depicting the relation between the four attachment quality types and internalizing problems and the other one depicting the relation between the four attachment quality types and externalizing problems.

#### 3.4.1 Internalizing problems and attachment subscales

**FIGURE 1:**
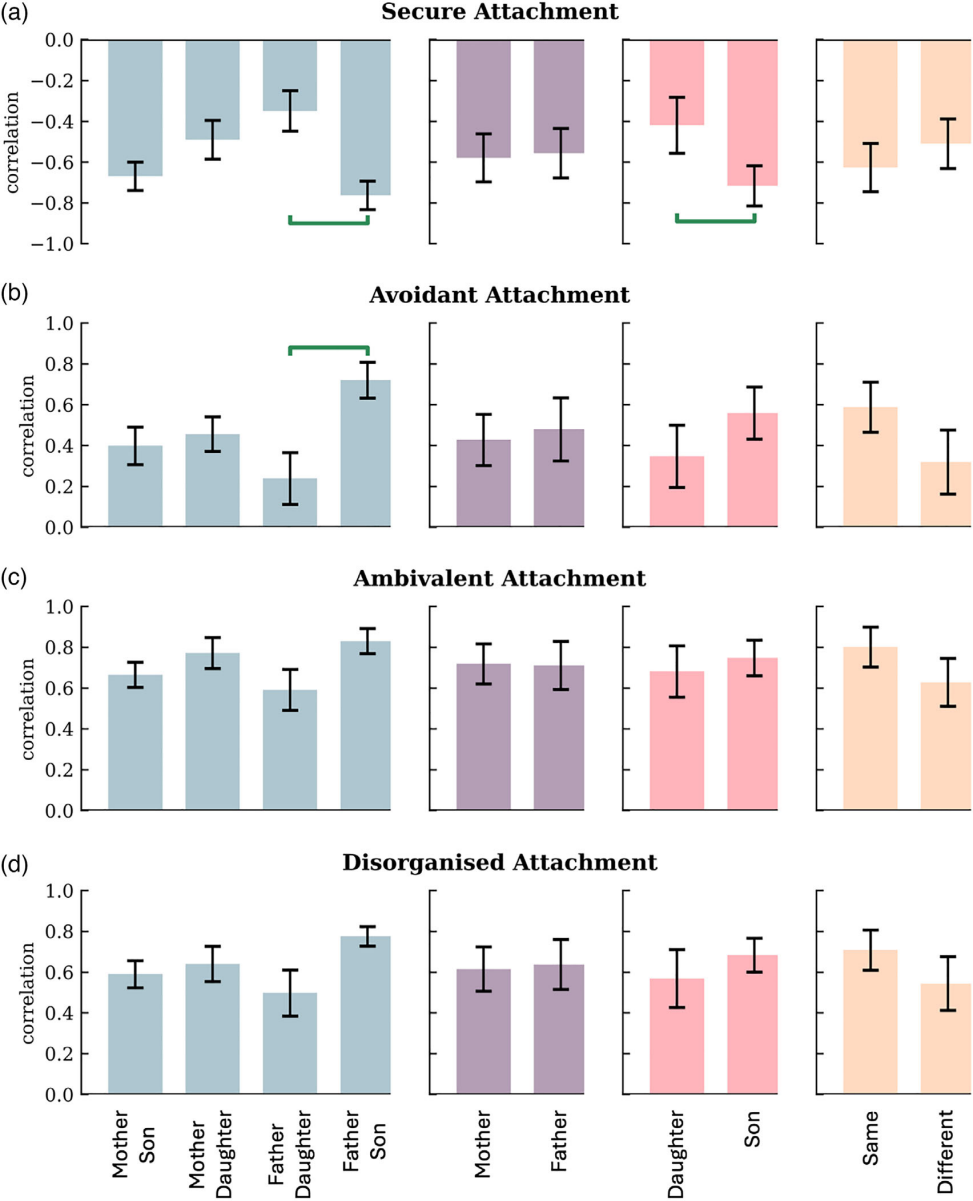
Gender moderation for internalizing problems and four attachment qualities. The bars represent the correlations between phantom latent factors per group. The horizontal green bars show significant differences in correlations, controlling for false discovery rate at *α* < .01.

When looking at internalizing problems, results regarding dyads showed a sizable difference in correlations between father–daughters and father–sons regarding the association between both secure and avoidant attachment. Specifically, in father–son dyads the negative correlation between secure attachment and internalizing problems was .414 units higher than in father–daughter dyads, *SE* = .12, *CI* = [.18, .65], *p* = .011 (Figure 1A), while the positive correlation between avoidant attachment and internalizing problems was .49 units higher than in father‐daughter dyads, *SE* = .15, *CI* = [−.78, −.18], *p* = .023 (Figure 1B). For the association between internalizing problems and both ambivalent and disorganized attachment, the results did not provide evidence for differences between dyads (Figure 1B,C).

Furthermore, results did not provide evidence for differences between mothers and fathers or between same and different gender pairs regarding each type of attachment and internalizing problems. With respect to child gender, a significant difference in correlations with secure attachment was found between sons and daughters, with sons’ correlation being .30 units higher than daughters’ correlation, *SE* = .08, *CI* = [.13, .46], *p* = .011 (Figure 1A). Regarding avoidant, ambivalent and disorganized attachment and internalizing problems, there was no evidence of differences between daughters and sons (Figure 1B–D).

#### 3.4.2 Externalizing problems and attachment subscales

**FIGURE 2:**
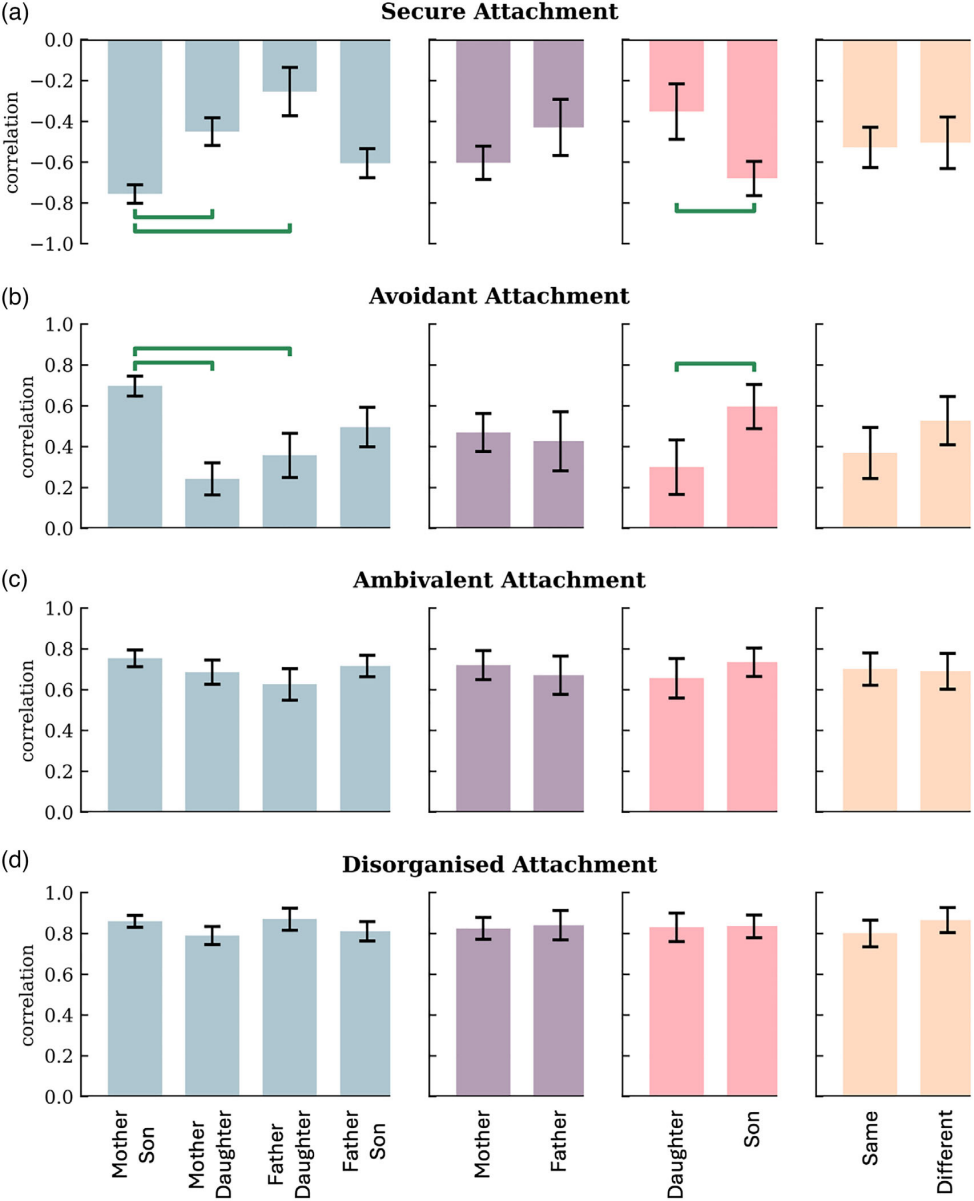
Gender moderation for externalizing problems and four attachment qualities. The bars represent the correlations between phantom latent factors per group. The horizontal green bars show significant differences in correlations, controlling for false discovery rate at *α* < .01.

When looking at externalizing problems, results regarding dyads revealed sizable differences in correlations between the mother–son and both father–daughter and father–son dyads regarding the association between externalizing problems and both secure and avoidant attachment. The negative correlation between secure attachment and externalizing problems was .306 units higher in mother–son compared to mother–daughter dyads, *SE* = .08, CI = [−.47, −.15], *p* = .011 (Figure 2A). The negative correlation between secure attachment and externalizing problems was .50 units higher in mother‐son compared to father–daughter dyads, *SE* = .13, *CI* = [−.75, −.26], *p* = .001 (Figure 2A). The positive correlation between avoidant attachment and externalizing problems was .46 units higher in mother–son compared to mother–daughter dyads, *SE* = .09, *CI* = [.27, .64], *p* < .001 (Figure 2B). The positive correlation between avoidant attachment and externalizing problems was .34 units higher in mother–son compared to father–daughter dyads, *SE* = .12, *CI* = [.11, .57], *p* = .024 (Figure 2B).

In addition, results did not provide evidence for differences between mothers and fathers or between same and different gender pairs regarding each type of attachment and externalizing problems. With respect to child gender, significant differences in correlations with secure as well as with avoidant attachment were found. For externalizing problems and secure attachment, sons’ correlation was .328 units higher than daughters’ correlation, *SE* = .08, *CI* = [.17, .49], *p* = .001 (Figure 2A). For externalizing problems and avoidant attachment, sons’ correlation was .30 units higher than daughters’ correlation, *SE* = .09, *CI* = [−.47, −.13], *p* = .004 (Figure 2B). Regarding avoidant, ambivalent and disorganized attachment and externalizing problems, there was no evidence for differences between daughters and sons (Figure 2C,D).

## 4 DISCUSSION

The aim of this study was to investigate the moderating effects of parents’ and children's gender on the association between levels of attachment quality (secure, avoidant, ambivalent, and disorganized) and problem behavior (internalizing and externalizing problems) in children between 2 and 5 years of age. The results suggest that the protective potential of attachment security and the risks of attachment insecurity could be different depending on the parent–child gender configurations. Differences are most prominent for secure and avoidant attachment, while for ambivalent and disorganized attachments, differences in the correlations are relatively smaller, not reaching statistical significance. The strongest relations are often seen in dyads with sons, but they differ depending on the parent gender and the nature of the problem behavior.

### 4.1 Results per grouping variable

Zooming in into gender differences at the dyadic level, distinct patterns of correlations emerge for internalizing and externalizing problems. For internalizing problems and all attachment quality types, the father–son dyad presented the highest correlation while the father–daughter dyad showed the lowest correlation in our sample. The difference between father‐son dyads and father–daughter dyads was the largest with respect to secure and avoidant attachment, reaching statistical significance. In contrast, differences regarding internalizing problems between mother–son and mother–daughter were smaller and did not reach statistical significance. This pattern partially matches Xu et al.’s (2018) findings that father‐son conflict predicts more internalizing problems, while father–daughter closeness and conflict do not predict problem behavior in preschool children.

For externalizing problems and both secure and avoidant attachment the mother–son dyad presented the largest correlation of the four dyads. The largest and significant differences were between the mother–son dyad compared to both father–daughter and father‐son dyads regarding secure and avoidant attachment. This contrasts Xu et al.’s (2018) finding that the mother–son closeness and conflict do not predict problem behavior. Differences between dyads in terms of problem behavior and ambivalent and disorganized attachment were smaller, not reaching statistical significance.

In sum, the results per dyad reveal a particularly strong association for both secure and avoidant attachment with internalizing problems for the father–son dyad, and with externalizing problems for the mother–son dyad. We speculate that the bi‐directional influence between internalizing problems and attachment quality for fathers and sons might be particularly strong because of a perpetuating cycle of fathers responding with avoidance to son's internalizing problems which in turn might accentuate children's internalizing problems as well as their avoidant behavior toward the fathers. This reaction pattern is in line with George et al.’s (2014) finding that kindergarten children show more avoidant attachment when their fathers are less responsive, but more ambivalent attachment when their mothers are less responsive. At the same time, a gender similarity effect might be stronger in dyads with fathers than with mothers. While mothers might facilitate similar amounts of self‐disclosure and might be able to identify internalizing problems and support their resolution equally well in sons and daughters, for fathers it might be easier to relate to sons compared to daughters. This might originate from the fact that fathers tend to spend more time with sons than with daughters, while mothers tend to spend equal amounts of time with their children (Raley & Bianchi, 2006). This might make a secure attachment with fathers a particularly strong protective factor for boys. Indeed, Starrels (1994) found in a sample of 6‐ to 11‐year‐old children that when it comes to closeness and nurturance mothers are equally involved with sons and daughters, but fathers are more involved with sons than with daughters.

Moreover, we speculate that the bi‐directional influence between externalizing problems and attachment for mothers might stem from mother's greater concern for the socially unacceptable behavior of boys (e.g., Hallers‐Haalboom et al., 2016), which might make son's externalizing problems more stressful, disrupting the mother–son relationship to a greater extent, which in turn might trigger more externalizing behaviors in sons. This is in line with Barnett and Scaramela's (2013) finding that mothers use less positive parenting with preschool sons than with daughters which in turn is associated with more externalizing behavior in sons compared to daughters. Furthermore, they are also in line with Gryczkowski et al.’s (2010) finding that lower levels of maternal positive parenting are more strongly associated with boys’ compared to girls’ externalizing problems in late childhood.

When we aggregated dyads by parents’ gender, we did not find any differences between mothers and fathers in the relation between attachment and their children's problem behavior. Dagan et al. (2021) also did not detect differences between fathers and mothers studying attachment and problem behavior in toddlers. At first glance, this matches the view that mothers and fathers have similar roles in children's upbringing (Grossman et al., 2008; Marshall, 2006) which perhaps is translated into similar contributions of their attachment relation to the development or the management of children's problem behavior. Yet, the results at the dyadic level do show that parents’ gender is involved, considering that the associations with secure and avoidant attachment were the highest in the father–son dyads for internalizing problems, while being the highest in mother–son dyads for externalizing problems. This matches Mathijssen et al.’s (1998) finding that mother–child relationship is and the father–child relationship is not associated with externalizing problems and El‐Sheikh and Buckhalt's (2003) finding that father–child attachment is and mother–child attachment is not associated with internalizing problems, but only for boys.

Concerning the gender effects of the child only, our results are in line with two meta‐analyses (Fearon et al., 2010; Groh et al., 2017) where the association between insecure attachment and externalizing problems was stronger for boys than for girls. There are at least two possible explanations for why this might be the case. First of all, the developmental mechanisms that lead to externalizing problems might include attachment to a larger extent in the case of boys than in the case of girls. The idea of different developmental paths to externalizing problems is supported by studies such as Hill et al.’s (2006) showing different factors associated with externalizing problems in boys and girls and Gardner et al.’s (2010) showing parenting interventions aimed at reducing conduct problems have a stronger impact on boys. Alternatively, despite sharing an underlying common mechanism toward externalizing problems, boys’ externalizing manifestations could be better captured by screening instruments, hiding the true extent of externalizing problems in girls (e.g., Chen, 2010). Additionally, we found a stronger association between secure attachment and internalizing problems for boys, as previously shown in the same age group by Madigan et al. (2013). These results match the hypothesis that boys are more sensitive to their environment than girls, especially in early childhood (Owens, 2016; Stinson, 1985). For example, Cooper et al. (2011) found that family instability is more strongly associated with internalizing and externalizing behavior in boys than in girls. Notably, our results cannot be generalized to age groups beyond early childhood. The moderating role of the gender of the child could change through development, becoming more pronounced for one gender or the other later on. This could explain why past literature has also revealed a stronger association for girls compared to boys between attachment insecurity and depression when the sample age range was broad (0–23 years old; Spruit et al., 2020).

Regarding gender similarity between parent and child, we did not find evidence for a similarity effect observed previously by Xu et al. (2018) and Hoeve et al. (2012). The similarity effect discussed by Russel and Saebel (1997) and Xu et al. (2018) predicts stronger effects of the quality of the parent–child relation on children's outcomes when parents and children have the same gender. While there was a pattern of stronger correlations for same gender dyads as compared to different gender dyads with regards to internalizing problems, the differences were not strong enough to reach statistical significance. It is therefore relevant to investigate these differences further with a larger sample.

### 4.2 Implications

Comparing the pattern of results we observe at dyadic level with the patterns observed when groups are aggregated by the parent's gender, the child's gender, or the similarity in gender, it becomes clear that a dyadic approach is more informative in order to paint a clear picture of the association between attachment and problem behavior. This calls for more studies which have a balanced number of mothers, fathers, sons, and daughters. Furthermore, our results show that the magnitude of gender moderating effects varies between the subtypes of attachment, motivating analyses that consider them separately. Given the presence of dyadic effects, parent–child gender interactions might be one of the factors behind the inconsistent results regarding attachment and problem behavior, because samples with a larger or smaller proportion of a certain type of dyad might result in the observation of larger or smaller effect sizes. Therefore, controlling for the parent‐child gender dyad rather than parent or child gender separately in between‐groups analyses is important.

The current study also provides implications for clinical practice. In understanding problem behavior of preschool children, assessing the attachment relationships with both parents is valuable. More specifically, we plead for involving all primary caregivers in this assessment and also taking into account the gender of the parent and the child in the attachment relationship. Especially for boys with problem behavior, the attachment relationships with their parents seem to be important factors. If further research establishes a causal relation between attachment and problem behavior, especially families with boys might benefit from an attachment‐based intervention to improve the attachment relationships and decrease problem behavior (Cooke et al., 2019; Mountain et al., 2017; Zeegers et al., 2020). Our study indicates that this might be the case, but our results are limited to demonstrating a bidirectional relation due to the cross‐sectional nature of this study.

## 5 LIMITATIONS AND SUGGESTIONS OF FURTHER RESEARCH

Our limited sample size (little more than 100 participants per parent–child gender configuration) as well as our stringent correction for multiple comparisons to control for false positives, means that we had a lower power to detect small gender moderating effects that might be present in the population. While our results indicate the presence of parent‐child gender moderation, future studies should evaluate the true extent of this moderation with considerably larger samples.

Furthermore, it might be that a good attachment relationship with one parent can compensate for the weak attachment relationship with the other parent (Louis et al., 2021). Recently, Dagan et al. (2021) showed that not one but two secure attachments protect children against internalizing problems, while one organized attachment is enough to protect children against externalizing problems. Including parent–child gender interactions in such studies will give us a more ecologically valid assessment of the moderating role of gender. In addition, having independent dyads was useful for disentangling the role of gender in the association between attachment and problem behavior, but in many families, there is more than one parent and often more than one child. While dyads in our sample did not significantly differ in the number of children present in the family, we did not have information about the gender of the siblings, nor did we have information about other caregivers who might be influential in children's life, such as grandparents. It is reasonable to believe that the family context and dynamics influence the association between attachment and problem behavior. It is therefore useful to speak of a network of caregivers and see how different attachment relationships interact to determine the children's outcomes (Dagan & Sagi‐Schwartz, 2021).

Parents in this study reported both about the quality of attachment with their children and their problem behavior. In early childhood, children spend most of their time with their parents, thus parents are important informants when it comes to their behavioral problems. However, the single‐informant approach might have introduced biases in the responses, such as inflating the association between attachment and problem behavior by adding common method variance (Reio, 2010). Most cautiously, the results should be seen as a reflection of parent's perception of the relationship with their children and their children's problem behavior. However, in the absence of previous evidence that some parent–child gender configurations are more affected by response biases than others, it is unlikely that response biases can account for the differences in correlations that we observe. Nevertheless, to fully mitigate the effect of bias arising from a same‐reporter design, observational measures, reports by other caregivers, or the children themselves should be employed in follow‐up studies. Furthermore, future studies could evaluate test‐retest reliability by dyad, to determine if there are differences in how reliable parents’ reports are based on the gender configuration of the dyad.

A potential confounding factor in our analysis is the time spent with the child, which was not measured in our sample. Because of indications from previous literature that parents might spend a different amount of time with their children based on their gender (Nikiforidis et al., 2018; Raley & Bianchi, 2006), future studies on the moderation of gender should strive to control for this variable.

Lastly, we are aware of having used a restricted view on gender, knowing that there is a considerable community of people who find that a non‐binary definition is more appropriate for their experience of gender (Richards et al., 2016). Adopting a more expansive definition of gender for both parents and children opens new avenues for future research.

## 6 CONCLUSION

In conclusion, our study shows that both the gender of parents and children can moderate the association between levels of attachment quality and problem behavior. The association between attachment and problem behavior is particularly strong for boys, but the extent of their internalizing problems most strongly matches the quality of attachment with their fathers, whereas the extent of their externalizing problems most strongly matches the quality of attachment with their mothers. The findings highlight the importance of considering gender dynamics when investigating the link between attachment and children's behavioral problems, because they demonstrate the merit of assessing the combination of parent and child gender. They also motivate research on possible differential results of attachment‐based interventions aiming to improve child behavior in different parent–child pairs based on gender.

## CONFLICTS OF INTEREST STATEMENT

Anouk Spruit was involved in the development and validation of the instrument “Attachment Relationship Inventory – Caregiver Perception 2–5 years (ARI‐CP 2–5)”. The psychometric analyses for that instrument were repeated in this article. Anouk Spruit is an employee of Basic Trust. The instrument ARI‐CP 2–5 is provided for free by Basic Trust. To prevent a bias in the interpretation of the results, she refrained from comments on the psychometric analyses for this instrument for the present study. The remaining authors have no conflicts of interest to declare.

## Data Availability

Full access to the anonymized dataset used in this study can be obtained upon request from the main author. The analysis code is available at: https://github.com/MagdaMatetovici/AttachmentProblemBehavior

## APPENDIX A

### A.1

In order to achieve partial measurement invariance among the four dyads with respect to the attachment latent factors, several modifications were necessary. This modifications were done by inspecting modification indices and Lagrange score tests (Bentler & Chou, 1993; Whittaker, 2012). The results presented in the paper are based on the SEM model which include these modifications. The residual covariance between the item “*My child turns away when I cuddle him/her.”* and “*My child ignores me when we see each other again at the end of the day.”* was set to zero for the mother‐son, mother‐daughter and father‐son dyads, while allowing it to be freely estimated in the father‐daughter group.

The loadings on the secure attachment factor for items: “*My child asks for my help when he/she needs it and lets me help him/her.”*, “*When I'm around, my child tries new things.” and “My child clearly shows me how he/she feels*.” were allowed to differ between the four dyads. The loadings on the avoidant attachment factor for items: “*My child just continues to play even after taking a hard bump*.” and “*My child runs off to see new things, without checking with me first.”* were allowed to differ between the four dyads. The loadings on the ambivalent attachment factor for items: “*I find it difficult to say anything when my child does something that I don't like.”*, “*Sometimes I feel so exhausted, that I cannot do anything for my child.”, “I feel like I can never do right by my child.”, “My child asks for help but is dissatisfied with what I offer.”, “My child needs my help with everything*.” were allowed to differ between the four dyads.

The residual covariance between the item “*Has at least one good friend.”* and “*Generally liked by other children”* was set to zero for the mother‐son, mother‐daughter and father‐son dyads, while allowing it to be freely estimated in the father‐daughter group.

The loadings on the internalizing problems factors for items: “*Has at least one good friend*.”, “Nervous or clingy in new situations, easily loses confidence” and “Picked or bullied by other children.” were allowed to differ between the four dyads.

**TABLE A1:** Thresholds, weak, and strong invariance attachment quality types.

Attachment type	Constrained thresholds			Constrained thresholds and loadings		
	$\Delta \chi^{2}$	*df*	*p*‐value	$\Delta \chi^{2}$	*df*	*p*‐value
Secure	23.66	18	.167	62.85	36	.004
Secure modified	‐	‐	‐	38.56	27	.069
Avoidant	54.60	34	.014	81.38	30	<.001
Avoidant modified	‐	‐	‐	34.07	24	.083
Ambivalent	37.38	33	.275	77.12	30	<.001
Ambivalent modified	‐	‐	‐	28.50	18	.055
Disorganized	33.39	42	.826	51.52	36	.045

*Note*: The level used to assess statistical significance was *α* < .01  In the first column, the configural model is compared to the constrained thresholds model. In the second column, the constrained thresholds model is compared to the constrained thresholds and loadings model.

**TABLE A2:** Thresholds, weak, and strong invariance problem behavior model.

Problem behavior	Constrained thresholds and loadings		
	$\Delta \chi^{2}$	*df*	*p*‐value
Internalizing problems	48.31	28	.01
Internalizing problems modified	28.49	19	.07
Externalizing problems	29.56	27	.33

*Note*: The level used to assess statistical significance was *α* < .01. The table shows the results of comparing the configural model to the constrained thresholds and loadings model.

**TABLE A3:** Approximate fit indices attachment models.

Attachment quality type	Configural invariance			Constrained thresholds			Constrained thresholds and loadings		
	CFI scaled	Gamma‐Hat scaled	SRMR	CFI scaled	Gamma‐Hat scaled	SRMR	CFI scaled	Gamma‐Hat scaled	SRMR
Secure	.929	.933	.099	.934	.918	.099	.937	.940	.117
Secure modified	‐	‐	‐	‐	‐	‐	.943	.945	.109
Avoidant	.794	.886	.121	.806	.928	.121	.765	.914	.138
Avoidant modified	.811	.930	.118	‐	‐	‐	.820	.933	.132
Ambivalent	.951	.956	.086	.954	.959	.086	.931	.940	.116
Ambivalent modified	‐	‐	‐	‐	‐	‐	.954	.959	.098
Disorganized	.947	.940	.086	.956	.950	.086	.960	.954	.101

Abbreviations: CFI, Comparative Fit Index; SRMR, Standardized Root Mean Square Residual.

**TABLE A4:** Approximate fit indices problem behavior model.

Problem behavior	Configural invariance			Constrained thresholds and loadings		
	CFI scaled	Gamma‐Hat scaled	SRMR	CFI scaled	Gamma‐Hat scaled	SRMR
Internalizing problems	.861	.945	.202	.885	.954	.216
Internalizing problems modified	.888	.955	.183	.895	.958	.205
Externalizing problems	.914	.928	.137	.934	.926	.147

Abbreviations: CFI, Comparative Fit Index; SRMR, Standardized Root Mean Square Residual.

## APPENDIX B ITEM AND FACTOR RELIABILITY

### B.1

For the ARI‐CP 2–5 and SDQ questionnaire we evaluated the item and factor reliability. The reliability for each questionnaire item in each dyad can be found in the online repository (https://github.com/MagdaMatetovici/AttachmentProblemBehavior), in the R objects “item_reliability_internalizing” and “item_reliability_externalizing”. Tables B1 and B2 show the factor reliability for each dyad, obtained with the R function compRelSEM() for the internalizing and externalizing models.

**TABLE B1:** Factor reliability attachment and internalizing problems.

Dyad	Secure attachment	Avoidant attachment	Ambivalent attachment	Disorganized attachment	Internalizing problems
Mother–son	.85	.72	.88	.93	.81
Mother–daughter	.84	.65	.81	.93	.79
Father–daughter	.80	.48	.80	.89	.64
Father–son	.80	.60	.80	.90	.74

*Note*: Values represent how much of the variance in the responses to the items is accounted for by each factor in the four different groups.

**TABLE B2:** Factor reliability attachment and externalizing problems.

Dyad	Secure attachment	Avoidant attachment	Ambivalent attachment	Disorganized attachment	Externalizing problems
Mother–son	.85	.74	.87	.93	.96
Mother–daughter	.85	.67	.80	.93	.90
Father–daughter	.80	.51	.79	.89	.75
Father–son	.79	.57	.80	.90	.90

*Note*: Values represent how much of the variance in the responses to the items is accounted for by each factor in the four different groups.
